# Managing buyer experience in a buyer–supplier relationship in MSMEs and SMEs

**DOI:** 10.1007/s10479-022-04954-3

**Published:** 2022-09-16

**Authors:** Prashant Kumar, Amit Kumar Kushwaha, Arpan Kumar Kar, Yogesh K. Dwivedi, Nripendra P Rana

**Affiliations:** 1 College of Business and Economics, Qatar University, Doha, P.O. Box 2713, Qatar; 2 Department of Management Studies, Indian Institute of Technology Delhi, New Delhi, India; 3 Emerging Markets Research Centre (EMaRC), School of Management, Room #323, Swansea University, Bay Campus, Fabian Bay, Swansea, SA1 8EN Wales, UK; 4 Department of Management, Symbiosis Institute of Business Management, Pune & Symbiosis International (Deemed University), Pune, Maharashtra India

**Keywords:** Buyer experience, Buyer–supplier relationship, Big data analytics, Small and medium enterprises, Social media, Machine learning, Text mining

## Abstract

Monitoring buyer experience provides competitive advantages for suppliers as buyers explore the market before reaching a salesperson. Still, not many B2B suppliers monitor their buyers’ expectations throughout their procurement journey, especially in MSMEs and SMEs. In addition, the inductive research on evaluating buyer experience in buyer–supplier relationships is minimal, leaving an unexplored research area. This study explores antecedents of buyer experience during the buyer–supplier relationship in MSMEs and SMEs. Further, we investigate the nature of the influence of extracted precursors on the buyer experience. Firstly, we obtain the possible antecedents from the literature on buyer–supplier experience and supplier selection criteria. We also establish hypotheses based on transaction cost theory, resource-based view (RBV), and information processing view. Secondly, we employ an investigation based on the social media analytics-based approach to uncover the antecedents of buyer experience and their nature of influence on MSMEs and SME suppliers. We found that buyer experience is influenced by sustainable orientation, management capabilities (such as crisis management and process innovation), and suppliers’ technology capabilities (digital readiness, big data analytical capability).

## Introduction

The COVID-19 pandemic caused an unprecedented global supply chain crisis, creating a considerable challenge for micro, small and medium enterprises (MSMEs) and small and medium enterprises (SMEs)^1^ to sustain themselves in the competitive market (Baral et al., 2021). Under such conditions, MSMEs’ suppliers face many operation challenges (Bianchi & Abu Saleh, 2020), disrupting demand fulfillment. So, the buyer–supplier relationship has immense value for MSMEs as intra-firm resources impact their power and operational balance (Kanyoma et al., 2018). However, the link between buyer and supplier is evitable and estrangement upon various issues (e.g., contract breach, transaction costs). Approximately 80% of business-to-business (B2B) leaders have changed their suppliers (Michaels & Doohan, 2019). In essence, MSMEs require a strategic plan for a long-term fruitful buyer–supplier relationship during an uncertain market environment. One such strategy is to improve the buyer experience, which indicates that buyers’ perception before and after selecting their supplier plays a critical role in providing suppliers with competitive advantages over others (Bianchi & Abu Saleh, 2020).

Furthermore, ensuring a better buyer experience impacts business functionalities such as increased satisfaction, and reduces the negative influence on supplier trust (Hawkins & Gravier, 2014) and communication elements between buyer and supplier (Claycomb & Frankwick, 2004), as well as contract-related details such as compliance with laws and regulations (Hawkins & Muir, 2014), behavioral and performance-based contractual complexity (Broekhuis & Scholten, 2018). Despite the advantage of buyer experience, there is a rift between buyers’ expectations and suppliers’ precedented understanding of buyers’ expectations in MSMEs with minimal resources and higher expectations from suppliers (Arend & Wisner, 2005). In addition, previous studies suggested a lack of research exploring buyer experience within the buyer–supplier relationship (Hawkins & Gravier, 2014), especially in MSMEs. There is a need for research that explores antecedents of buyer experience within buyer–supplier throughout the suppliers’ touchpoints. However, many MSMEs lack the resources to conduct a broad survey or market research to know their suppliers and buyers (Alshawi et al., 2011). Therefore, a solution necessitates minimal resources and a financial budget. Also, analytical capabilities provide MSMEs with a pathway for success and innovativeness by empowering their information-accessing capabilities. Looking towards a need for buyer experience research, we have formulated our research questions as follows:

*RQ1: What antecedents impact buyer experience during buyer–supplier encounters in the MSME sector?*

*RQ2: How do these antecedents impact buyer experience during buyer–supplier service encounters in the MSME sector?*

Since MSMEs face competition in terms of their product supply and have a challenge regarding resources, under such conditions the resource-based view (RBV) theory is the suitable choice to access the buyer experience or to gain a competitive advantage. To analyze these research questions, we use an inductive research approach. We first extract the factors associated with buyer experience from supply selection criteria and buyer–supplier literature, then develop hypotheses for model validation. Such an inductive approach helps to overcome saturation in theory in an established area of research (Kar & Dwivedi, 2020). We adopt a social media (SM) method for data collection and extraction of antecedents. The conversational exchange of experiences (Kushwaha & Kar, 2021) by buyers and suppliers of MSMEs posted on neutral social media platforms (SMP) such as Twitter contributed to creating user-generated content (UGC). Furthermore, the unbiased signals (themes or text clusters) are captured through exploration and assigned to theoretical factors in line with antecedents tested on their significance for driving the buyer experience through the lasso regression method.

Further, we contribute to literature on buyer experience during buyer–supplier relationships by extending factors associated with three themes based on RBV theory: management capabilities, technology capabilities, and sustainable orientation. Also, we explain the nature of the influence of factors related to these three dimensions on the buyer experience. In addition, we implement a more robust topic extraction method by implementing Latent Dirichlet Allocation (LDA)-based topic modeling on top of the hierarchical clustering approach.

From this point forward, the paper progresses as follows: Sect.2 represents the literature review of buyer experience in MSMEs. Section3 describes the methodology and data collection procedure adopted for this study. Section4 presents the exploratory analysis of collected data, followed by Sect.5, which provides the confirmatory analysis. Section6 presents the discussion, implications, and limitations. Finally, Sect.7 concludes this study.

## Literature review

This work extends previous research on customer experience (CX) and service operations, which argues that CX (buyer experience in this work) is essential for gaining a competitive advantage in the market. However, this study adopts an inductive approach to extract factors from existing buyer experience research relevant to the buyer–supplier context. To elaborate on the context first, we discuss the role of CX in maintaining an effective buyer–supplier relationship. Following this, we explain the importance of the buyer–supplier relationship in MSMEs. Lastly, to set the methodological context to extract the buyer experience information, we discuss the different technical solutions to measure buyer experience.

### Buyer experience for improving the buyer–supplier relationship

Based on various contexts (pre-, present, and post-engagement interaction), literature indicates buyer experiences differently. For example, some researchers believe accumulated knowledge or understanding is gained by a buyer from prior buyer–supplier interaction (Hawkins & Muir, 2014). Some researchers consider buyers’ hedonic and utilitarian experiences during the buyer–supplier relationship (Broekhuis & Scholten, 2018). Experience comes from the manager’s tendency to apply tacit knowledge based on prior buyer–supplier interactions (Hawkins & Gravier, 2014), pointing towards management quality. Further, looking from supplier perspectives, understanding the buyer characteristics helps suppliers understand the building blocks of the relationship (Claycomb & Frankwick, 2004); in essence, it signifies customer orientation (i.e. considering customers’ needs first).

Considering sustainable competitive strategies and predictive outcomes, RBV indicates a framework incorporating organizations’ intangible and tangible resources (Kozlenkova et al., 2014; Zahra, 2021). Given that MSMEs have scarce resources and various commitments, Zahra (2021) indicated RBV as a convenient framework for accessing resources that may help with gaining competitive advantages. Managing functional and buyer experience requirements may be difficult; still, the strategic advantage of improving buyer experience makes 39% of MSMEs consider it a top priority, even more important than raising profits or cutting expenses (Moyo, 2020). MSME literature considers buyer experience as a cause of measured phenomena or a mediating variable between two variables. For example, experience facilitates entrepreneurs to seek inter-firm ties and the involvement of suppliers in the product development process (Lipparini & Sobrero, 1994); buyer experience is a predictor of disengagement behavior during buyer–supplier contract conflict (Mpeera Ntayi, 2012); experience is a moderating variable between buyers’ perceived cultural similarities and relationship commitment (Bianchi & Saleh, 2020).

Conversely, the literature indicates the importance of buyer experience in success and improved firm performance (Adams et al., 2012; Feng et al., 2021). However, the literature review found that research on buyer experience assessment for sustainable buyer–supplier is shallow. So, we formulated our first question: which criteria link with buyer experience in MSMEs, as finding these major themes (discussed in the following subsection) help to organize buyer experience more efficiently? Therefore, there is a need to extract the factors influencing the buyer experience, which is not yet tracked despite the importance of buyer experience.

The second question regards the nature of the impact, such as how the extracted factors influence buyer experience. This is important for suppliers’ MSMEs to gain competitive advantages in the market, which we cover in this study.

So, firstly we need to explore which resources (organizational, financial, human, and physical) have a higher impact on gaining a better buyer experience. In this context, Kozlenkova et al., (2014) indicated using the VRIO lens of RBV incorporating valuable, rare, and inimitable organizational resources confer strategic benefits. Extending these perspectives, we borrowed from supply chain literature to further elaborate on the buyer experience and its associated factors. Visiting the sustainable suppliers’ management, buyers select the suppliers based on various criteria such as financial performance, external perception, service capabilities, commitment (Igarashi et al., 2013; Zimmer et al., 2016), technological capabilities, flexibility, innovation (Kar & Pani, 2014), and collaborative routing (Konstantakopoulos et al., 2021).

### Managing buyer–supplier relationships for MSMEs

The buyer–supplier relationship involves different marketing and exchange activities between two parties. Especially for MSMEs, which face many constraints in their business activities, their resource occupation strategies include outsourcing from multiple sources, where larger firms mostly choose a single source. The main question, therefore, is how MSMEs maintain better buyer–supplier relationships to strengthen their business functionalities (Mudambi & Schründer, 1996; Tam et al., 2007) where the connection deteriorates due to different reasons such as their individual expectations or opportunistic behavior (Adams et al., 2012), or the influence of economic and social factors on the ex-post transactional cost (Shahzad et al., 2018). Transactional cost and relationship commitment are central ingredients in maintaining a sustainable buyer–supplier relationship in MSMEs. So, the above argument points towards a better execution capability for a fruitful buyer–supplier relationship. Considering the RBV’s central construct, capabilities as “an organizationally embedded non-transferable firm-specific resource” (Kozlenkova et al., 2014, p.5) increase productivity by deploying stretched resources more efficiently. So the *management capabilities* of SME managers help in cost-saving, responsiveness, and contract management as many MSMEs face limited capacities and resources, increasing competitive and unfavorable business environments, and the creation of operations supply risk (Chowdhury et al., 2019).

Sometimes, the buyer–supplier relationship for MSMEs depends on demographics, awareness, and business heterogeneity. The small business’s procurement behavior, nature, size (Morrissey & Pittaway, 2006), and cross-cultural relationships (Andersen et al., 2009; Cooray & Ratnatunga, 2001) influence the buyer–supplier relationship. However, the association is derived from trustworthiness, quality (Bianchi & Abu Saleh, 2020), communication linkage, planning horizons, understanding of effort-reward or requirement of each entity, and the effective use of link-pin (Thakkar et al., 2007). So, broadly specifying these features is associated with the stockholders present during the buyer–supplier relationship. So, we consider *stakeholder’s orientation* a critical facet of the sustainable buyer–supplier relationship in MSMEs.

Furthermore, real-time interactivity and information exchange in the supply chain and buyer–supplier relationship influence MSMEs to adopt new technological solutions (Deepu & Ravi, 2021; Ghobakhloo et al., 2011). For example, using Information and Communication Technologies (ICT)-enabled supply chain interaction (Mirkovski et al., 2016) or electronic markets (Scuotto et al., 2017) as facilitators in the buyer–supplier relationship. However, while they benefit from technology-enabled value-added participation and increase the reach of suppliers, the coordination cost during supply chain management is high (Mirkovski et al., 2019). So, *technical capabilities* help MSMEs improve their performance, though they need to consider environmental uncertainty before integrating technical solutions.

**Fig. 1:**
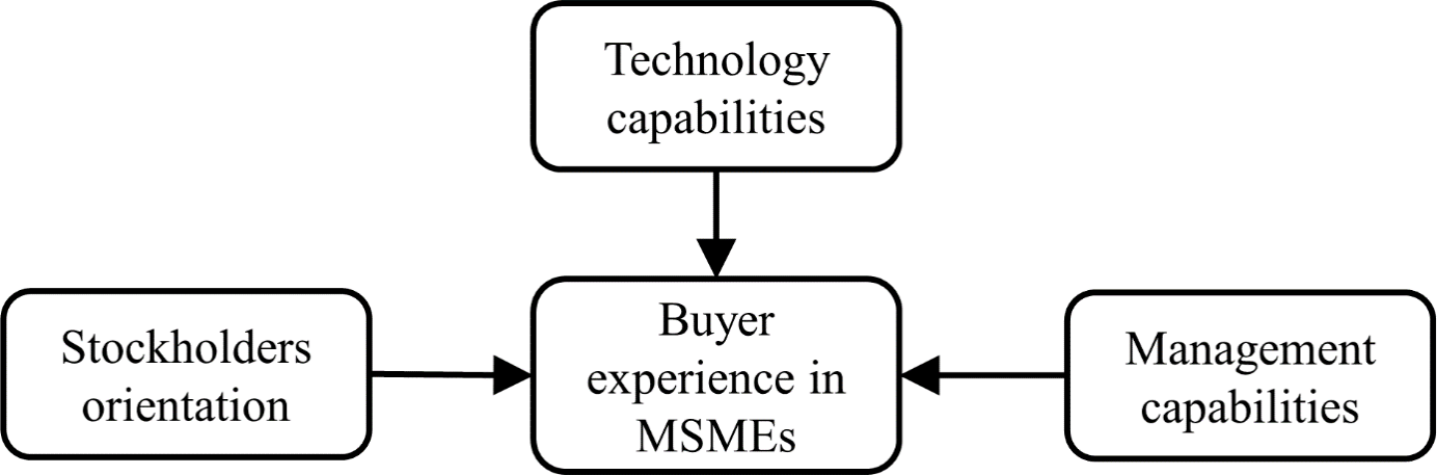
Themes associated with buyer experience in the literature

Moreover, management capabilities improvise the selection of resources; stakeholders’ orientation makes them categorize buyers, and technology helps MSMEs reduce their communication gaps; understanding customers’ preferences before making any major decision can establish a better buyer–supplier relationship (Lancastre & Lages, 2006). Looking at the broad literature, we analyzed those studies linked with the central themes shown in Fig.1. Moreover, these studies focused on quantitative aspects relating to management capability, technical capabilities, and sustainable orientation with SMEs’ success or performance. However, these themes affect MSMEs’ buyer–supplier relationships has been studied significantly less. In essence, understanding how elements of these three themes influence buyer experience is essential for MSMEs, especially when considering a long-term sustainable buyer–supplier relationship.

### Prior literature on the approach used to access the buyer experience by MSMEs

During our investigation, we did not find any study to assess buyer experience in MSMEs. So, we borrowed the methodology to measure buyer experience in buyer–supplier and the supply chain literature. We analyzed that most studies utilize the survey (Claycomb & Frankwick, 2004; Hawkins & Gravier, 2014; Hawkins & Muir, 2014) or case study (Broekhuis & Scholten, 2018) to extract the buyer experience. Besides traditional methodologies, many researchers pointed toward the potential use of available data and analytical capabilities in supply chain management (Chae, 2015). For example, we accessed customer dynamics (Ram & Zhang, 2021) and the challenges and criticality of SM and analytics in the supply chain (Choi et al., 2020). A few researchers considered utilizing SMA to build strategies to manage the supply chain (Mishra & Singh, 2018). Besides, SM literature for MSMEs suggests branding strategies to improve buyers’ satisfaction and confidence (Hsiao et al., 2020). A few studies suggest the super-additive value of SMA in functional complementary in MSMEs (Dong & Yang, 2020).

Furthermore, SM platforms and big data analytics have been extensively used in various streams such as disaster management (Akter & Wamba, 2019; Kavota et al., 2020), service operations (Wamba et al., 2019), and organizational sensing capabilities (Wamba et al., 2020), to reduce the time complexity of collecting data. Looking towards such extensive use of SM and SMA in supply chains and MSMEs, this study bridges the gap of connecting buyer experience factors from the data generated by users on an open platform. MSMEs can cost-effectively avail the buyer experience information.

## Research Methodology

**Fig. 2:**
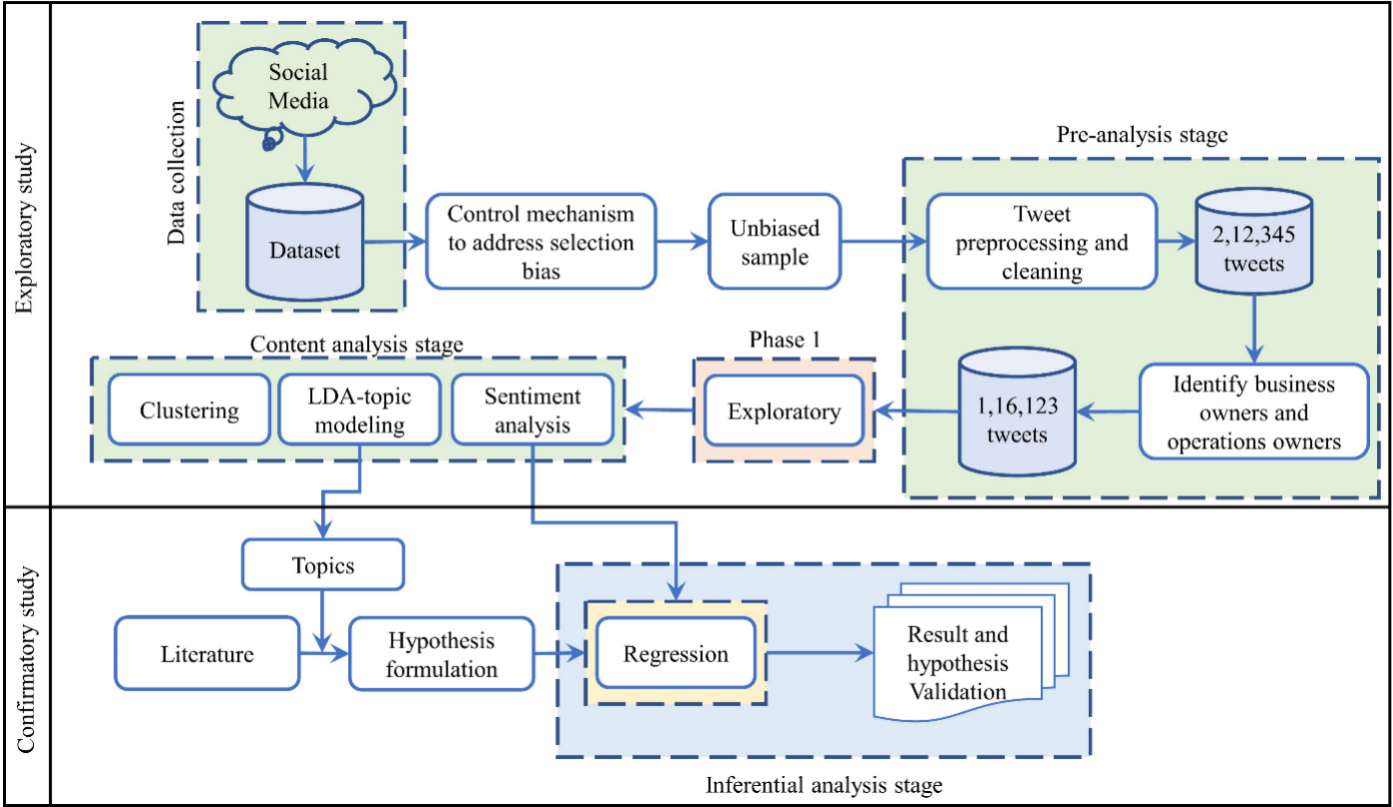
The methodology selected for this study

For the analysis methodologies, we followed the analysis methodology suggested by Berente et al. (2019), indicating the data-driven theory-building approach, which incorporates four stages: ‘sample observation’, ‘generate a taxonomy’, ‘identify qualitative and quantitative relationships’, and ‘generate structural and process models’. For this study, we undertake an SM data-based unbiased inductive learning approach (Kar & Dwivedi, 2020; Liu et al., 2021) in which we first drive the factors from the literature; with by the help of data, we further shape the factors closely interlinked with buyer experience. We present the framework used for this study in Fig.2. We select Twitter for data collection, allowing owners of all-scale organizations (start-ups to large-scale production houses) to share their buyer and supplier experiences. At times, these owners even tag specific operation partners (delivery partners, supplier partners) to advocate and share good experiences and, on the other hand, share bad experiences for another owner to learn.

### Data collection

We considered extracting the tweets using the hashtags related to SMEs and MSMEs based on the suggestion of Oh et al., (2015). They indicated that data from hashtags indicate all thematically relevant tweets, represent the multifaceted views, and can be used to access the collective behaviors. Initially, we collected around 10,000 tweets and analyzed data signals to understand the trending topics related to operation analytics in MSMEs worldwide. This exercise objectively bestowed 10 top trending operation analytics hashtags, which were controlled for MSMEs by filtering only for the hashtags that co-occur with #SME and #MSME, the first data control mechanism.

We proceeded to actual data collection with the imperative data collection strategy as part of the inductive framework’s first step. We collected the UGC data (tweets) from January 2020 till August 2020. We executed the data collection scripts on the Python tool using the streaming tokens of Twitter. This exercise gave us a sample of 347,145 tweets posted by 59,451 users. We then deployed the second data control mechanism to remove the marketing and promotional tweets, followed by the tweets posted by bots, to drop the unnecessary noise from the data and ensure that the inferences were drawn from the data unbiased.

The raw UGC data contained much noise in terms of special characters, HTML, and URLs, which had to be cleaned. Tweets posted from automated bots and major official accounts that had followers and were following greater than 10,000 other accounts were dropped from the dataset to ensure there was no structural bias and low promotional content in the data. Finally, we used stemming and lemmatization to arrive at the root words giving us the right sentiment and emotion from a text (Paice, 1994). This control mechanism was fine-tuned by designing a key that was a combination of the hour when tweets were posted, the number of tweets posted each day, and the overall volume of tweets, to further ensure that the unwanted noise was dropped from the data. The collected sample contained accurate signals on the MSMEs. At the end of this exercise, the resulting sample size was 212,345 tweets posted by 52,707 users.

Besides, social media data faces challenges in terms of reliability and validity because data generated from crowdsourcing may contain fake information. We deployed the third control mechanism to create a specific sample by restricting user accounts from which the tweets were posted. The list that was used to limit the accounts had designations such as ‘head’, ‘owner’, ‘line head’, ‘vertical head’, ‘supply head’, ‘supply manager’, ‘delivery manager’, ‘logistics manager’, ‘operations manager’, and ‘operations head’. This mechanism helped us to be unbiased and reliable, yet only appropriate user-generated data were created by operations decision-makers in respective organizations on active MSME topics. The final sample for the analysis was reduced to 116,123 tweets posted by 47,135 users. Here, users are unique users extracted by considering a user counted only once, regardless of how many times they tweeted in the final 116,123 tweets.

## Study 1: exploratory analysis

### Content analysis

The text-mining-based content analysis method (Kumar et al., 2021; Kushwaha et al., 2021a, b) consists of four unique steps to uncover truthful signals within social media conversations. In the first step, we start the content analysis by calculating each cleaned tweet’s polarity and subjectivity score (de-noised from the previous step) (Chae, 2015) using the Stanford Natural Language Processing Library, as it is updated constantly and represents fine-grained sentiment labels for words, sentences, and phrases. The Python tool provides open-source libraries such as scikit-learn to achieve this task by taking cleaned tweets as inputs and generating various output scores. These scores can help classify the tweets into negative, positive, and neutral sentiment groups. We summarize the data algorithmically in the second step to understand the prominent and frequently occurring topics. We use Latent Dirichlet Allocation (LDA) (Tirunillai & Tellis, 2014), also known as topic modeling, for text summarization. This text summarization step also helps inductively uncover the data’s inherent themes of operation analytics. We identify the factors supported by operations management theories based on these themes. The LDA model developed at this stage consists of 20 words to summarize the overall data better.

In the third step of content analysis, we now create a network diagram of the LDA model topics to learn the words’ co-occurrence through a visual representation. The topic network diagram applies network science, which treats the issues as nodes and the relationship among these topics as edges connecting these nodes. The number of connecting edges represents how strongly a topic co-occurs with the rest of the model’s topics and helps create a community of topics. Furthermore, deploying the inductive learning approach, this community diagram allows us to validate a few assumptions and hypotheses as a simple correlation among the topics by visually validating if the topic co-occurs. We first create a network diagram at an overall level and deep-dive into understanding the signals from data; we repeat the same exercise for the respective sentiment groups created in step 1 of the content analysis. As part of the fourth step of content analysis, the co-occurrences of the topics identified from LDA are extended to establish each topic’s co-occurrence with other words in the respective sentiment groups. This is achieved through the decision tree approach, which shows the relationship between factors (Brusco et al., 2012).

For the validity, we incorporate a two-stage process, in which first we include LDA, which provides topics and their associated words. These words are then mapped with the help of WordNet, a lexical database of English (Fellbaum, 2005), for a better representation of each topic, leading to a valid representation of topics and their associated words (Alkhodair et al., 2018). As for the reliability, we employ inter-coder reliability, in which one author has assigned each topic to its closely associated constructs, followed by other authors who mark the given relations. Lastly, we calculate the inter-coder reliability score, which is found to be 0.87, greater than the 0.70 criterion indicated for exploratory research (Rust & Cooil, 1994).

### Results

**Fig. 3:**
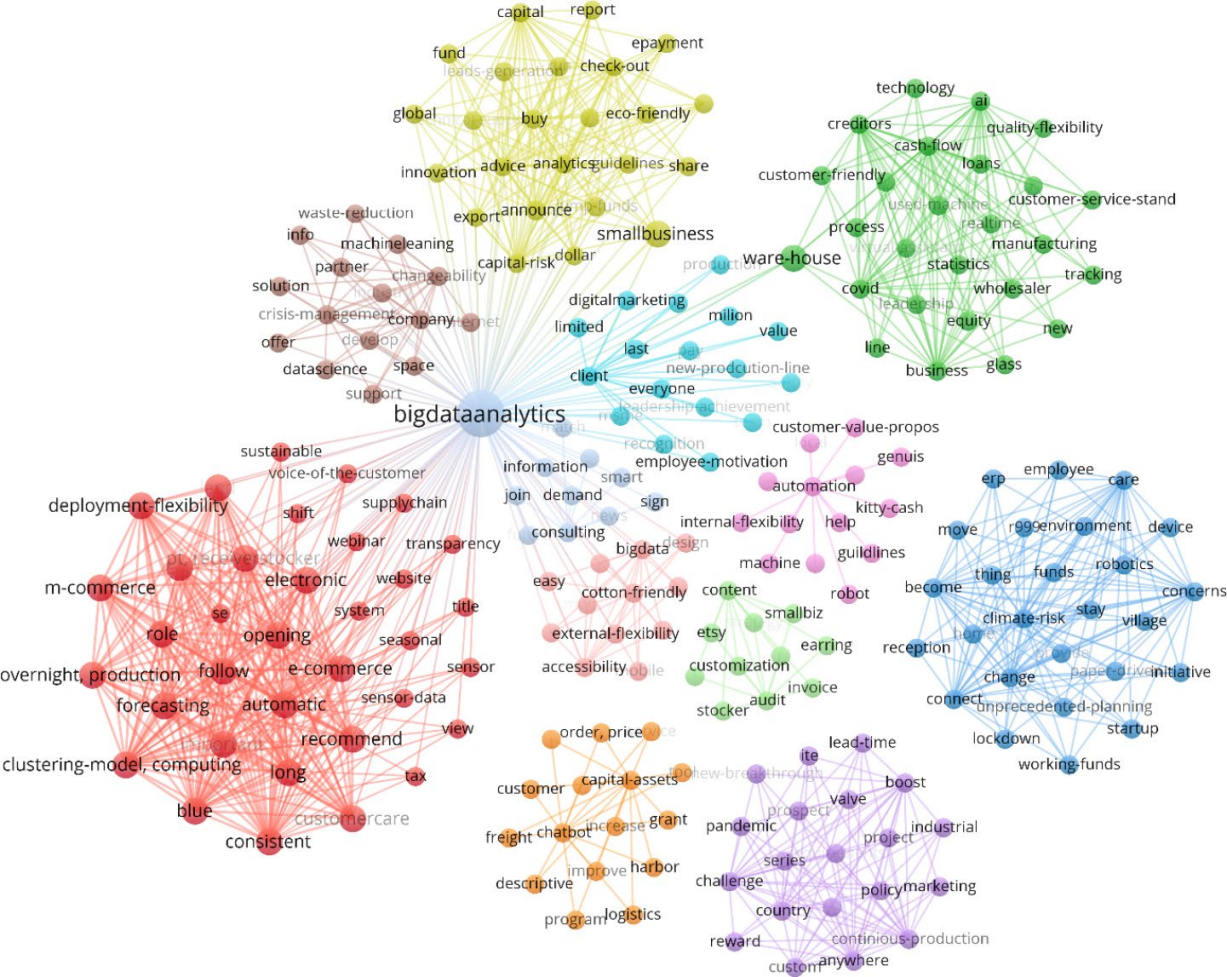
45-Topics and association between topics extracted using Latent Dirichlet Allocation

Firstly, we applied topic modeling using LDA to extract the optimal number of themes or factors. In our dataset, we found 45 optimal topics that could be extracted without getting similar words in two or more topics. So, a network diagram of terms present in 45 topics is presented in Fig.3. The network diagram helps cluster the associated themes into closely occurring factors. Literature and theories must address these factors to connect with the research objectives. So, Fig.3 highlights all the topics with their words, and if one tries to look back into existing literature to connect these factors with likely names, possible constructs could be defined. For example, a word in the topic such as ‘customer-value-propose’, ‘help’, or ‘guidelines’ (light green color in Fig.3) is related to customer-specific factors, so when we look at the dataset samples from which these words are mapped, we find these words fall in customer-orientation-related factors.

Similarly, words like ‘reward’, ‘employee’, ‘consulting’, ‘friendly’, and ‘respond’ (dark green color) incline more towards the employee-specific entity. Therefore, we have mapped these words to employee orientation. All extracted topics are mapped through this process, with 14 factors related to buyer experience within a buyer–supplier relationship in MSMEs. Lastly, we attempted to identify the factors through topic association to formulate the relationship with existing literature and theories. A team of two researchers also assessed 20 random snippets of the dataset independently during this process of topic mapping for all 14 factors to ensure content and linguistic validity.

**Fig. 4:**
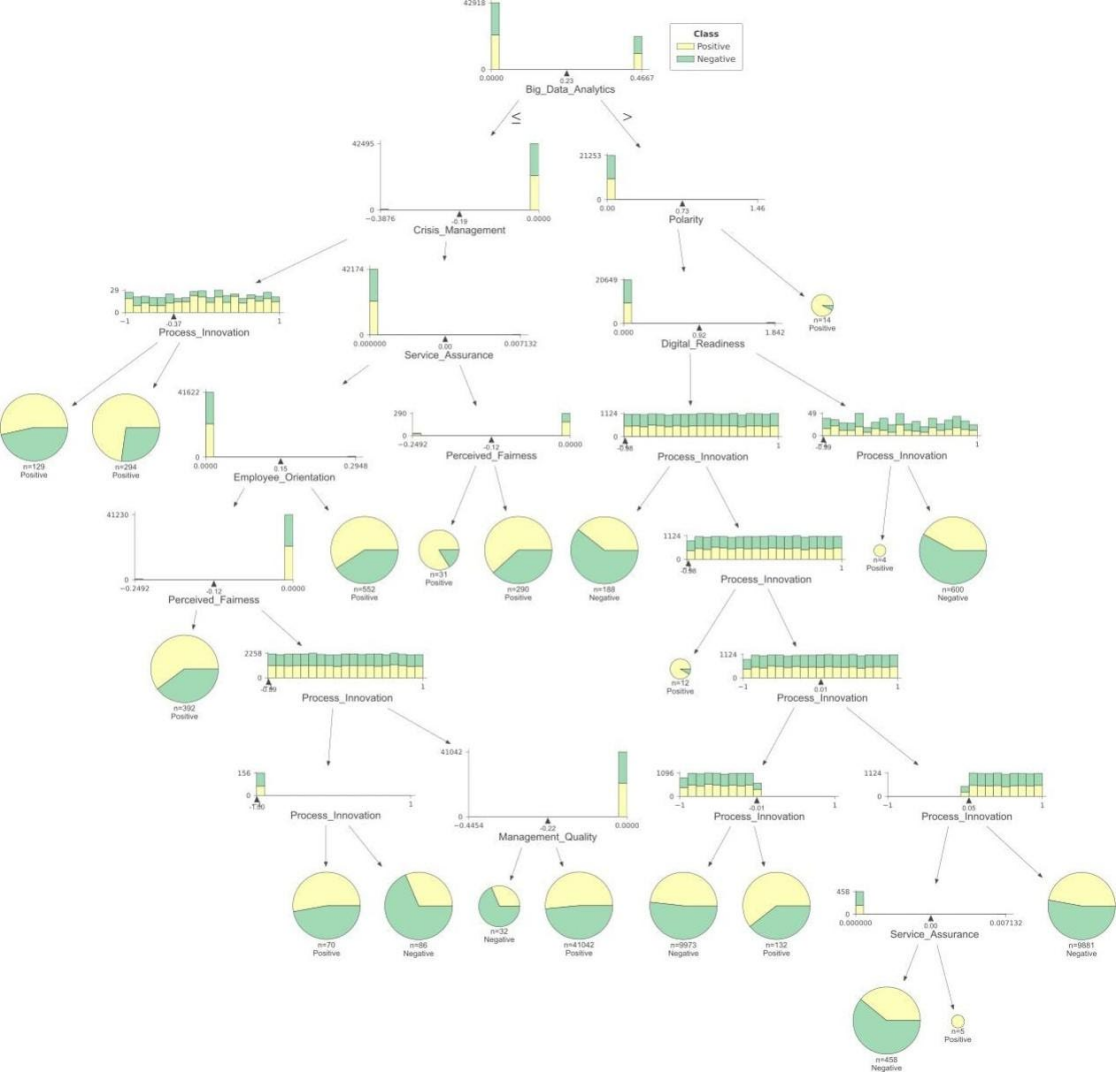
Decision tree showing the evolution and relationship between factors

Secondly, we have presented the result of the decision tree in Fig.4, which shows the decision points and a histogram illustrating the distribution. Using a decision tree, an interpretable model can be analyzed efficiently, as presented in Fig.4, indicating that big data analytics is the starting point of the tree from where branches have been formed further.

## Study 2: confirmatory analysis

Once we complete the four steps of content analysis, starting with summarization and ending with clustering of the words in each sentiment group, we can establish the theoretical factors that have emerged through an inductive analysis method without bias. Operation theories back these factors; however, we must validate these factors (hypotheses) through inferential data analysis. Traditional exploration methods of the inductive learning of signal capturing, if not tested, might have a scope of error when used by management scholars for theory building, as many relationships may emerge, which otherwise may not be statistically significant.

Lasso regression is the statistical method to validate the current research hypothesis. The linear regression analysis examines the connection between the dependent and estimated variables while minimizing gradient descent, such square residuals. Conversely, in the big-data case, the nonlinear technique outperforms the linear method (Hastie et al., 2009). As a result, nonlinear approaches such as lasso might aid in solving the variable optimization procedure (Varian, 2014), as their analysis focuses on standardizing all non-constant predictors to get a mean of 0 and a variance of 1. The sum of the residual squares with the regularization term is minimized to limit other factors’ influence. The lasso regression approach reduces the most negligible square regression value to 0, making the statistical process more efficient and viable.

### Hypotheses development

Initially, inductive research starts with observations and identifying the pattern in the dataset, followed by prior theories and observation, which helps develop new approaches. Based on the content analysis of SM data, we have observed 14 factors associated with buyer experience in MSMEs, further categorized into four different themes. In addition, we elaborated on the relationship between these factors and buyer experience based on transaction cost theory (TCT), RBV, and information processing theory (IPT). We used RBV to build the framework consisting of stakeholder orientation, management capabilities, and technical capabilities, followed by IPT and TCT to reorganize the factors and establish the relationship with the buyer experience. Based on these 14 factors and literature, we have argued 14 hypotheses.

#### Stakeholder orientation

IPT incorporates the organizational strategies of using information-processing capabilities to gain competitive advantages and deliver exemplary service at the right time. Information sharing and utilization are directly correlated with customer orientation, mediating the relationship between future customer information sharing and purchase/repurchase behavior (Ghouri et al., 2021). Besides, VRIO assumes that SMEs have resources that will be touchpoints from which the firm can gain information. So, the manager needs to consider their customers’ mindset, employees, and sustainable performance opportunities to better access their experience.

Customer orientation (CO) specifies the strategy where suppliers deeply understand buyers’ preferences and expectations (Shin et al., 2000). So, creating a CO strategy is advantageous for SMEs compared to larger firms due to restricted and localized buyers in a considerably shorter line of contact. Further, Jones & Rowley (2011) found that close interaction relationships facilitate benefits such as buyers’ loyalty and satisfaction. In addition, SMEs’ quick response to customer queries is key to establishing a long-term relationship between the SMEs’ suppliers and customers. Furthermore, the expectancy theory (Laffineur et al., 2020; Manolova et al., 2007) suggests that one’s behavioral choice when making any decision is a function of expectation about fulfilling their desired objective. If a SME’s buyer feels redundancy in fulfilling their goal, they will move toward other suppliers. We have also observed that users use words associated with customer orientation for a better experience in the dataset. We argue that a supplier could provide a better buyer experience by maintaining a better customer orientation. Hence, we formulate our first hypothesis as follows:

##### H1

Customer orientation is positively associated with buyer experience in MSMEs.

Literature suggests that many MSMEs lack human or skilled human resources due to financial capabilities or other constraints (Kim et al., 2006). In many cases, MSMEs face the challenges of dealing with their buyers during the contract negotiation or providing optimal customer service. So, employee orientation (EO), which represents the organizational process of nurturing employees for their business functionalities, plays a significant role. EO helps firms achieve better performance by enhancing trust between themselves and employees, resulting in lower employee turnover (Vaitoonkiat & Charoensukmongkol, 2020). Especially from SMEs’ perspectives, EO helps them reduce the constant search for skilled employees and helps them preserve firms’ knowledge, behavior towards customers, and extra financial burdens (Caputo et al., 2021). Besides, core customer experience literature suggests that an organization with a skilled customer touchpoint employee provides a better customer experience. So, we have formulated our next hypothesis as follows:

##### H2

Employee orientation positively influences buyer experience with MSMEs.

Sustainability contributes significantly to the economic development of countries and MSMEs (Malesios et al., 2020). Sustainable orientation (SO) infers “the overall proactive strategic stance of firms towards the integration of environmental concerns and practices into their strategic, tactical, and operational activities” (Roxas & Coetzer, 2012, p.464). The highly sustainable supplier improvises their efficiency through product innovation (Abdelaziz et al., 2020). Their employees are oriented towards new product designs, unique product attributes, and manufacturing processes (Roxas & Coetzer, 2012). On looking at these initiatives within supplier firms, buyers’ firms sometimes get motivated to implement sustainable orientation into their operations, further affecting the buyer experience. So, our next hypothesis would be as follows:

##### H3

Sustainable orientation positively influences buyer experience with MSMEs.

#### Technological capabilities

RBV studies highlight VRIO’s ongoing advantage, suggesting enterprises have flexibility when picking and deploying resources. MSMEs lack credibility and do not always have the most significant functional capabilities. They may purchase equipment from failed firms and personalize it based on their ‘make do’ strategies with little resources (Zahra, 2021). However, the cognitive roots of inventiveness become a deliberate resource that helps MSMEs bypass conventional resource limits. So, technological innovation could allow MSMEs to access the global market and overcome competitiveness. While investigating multiple discount problems, Muk & Chung (2015) found that technological innovation helps suppliers provide their buyers with pre-announced price cuts and an existing product at lower costs. However, customer experience research suggests that buyers’ choice to utilize a technologically innovative solution such as artificial intelligence adoption (Grover et al., 2020; Kar & Kushwaha, 2021) for demand forecasting depends on their expertise in using technology. Like buyers with better digital readiness (DR), they are more susceptible to innovative technology than those who are not experienced. Therefore, we hypothesize:

*H4: Digital readiness is positively associated with buyer experience during the buyer–supplier relationship in MSMEs.*

In a crisis or pandemic situation, technological innovation (TI) (Tan et al., 2017) in the supply chain provides a breakthrough path for buyers and suppliers. Besides, when the relationship moves towards the long-term and corporate, both parties must frequently coordinate, especially when adopting innovative technologies (Hausman & Stock, 2003). However, considering the limitations (lack of skill, financial constraint in implementing advanced technology) faced by MSME’s buyers, sudden changes in supplier strategies for adopting technological solutions may create a challenge for buyers. So, the risk and benefits of implementing technical innovation overlap and must be balanced against one another. So, we propose our hypothesis as follows:

*H5: Technological innovation may positively influence buyer experience with MSMEs.*

Information retrieval and sharing explain the buyer’s perception of the supplier’s ability to provide information accurately (Yang, 2014). We have previously argued that suppliers must fulfill demand during a crisis. So, predicting the impending crisis could help SME suppliers to manage the resources for orders effectively. However, the solution of the product needs to be accurate and up to the optimal level. So, big data analytic capabilities (BDAC) within firms could help organize the information and intelligence for various purposes such as demand forecasting and innovation. Customer satisfaction research suggests that reliability and associated factors such as homogeneity and test time affect customer satisfaction (Kar, 2020), influenced by information-retrieving capability, further impacting customer experience. So, we hypothesize:

*H6: Big data analytics capability positively influences buyer experience with MSMEs.*

We found many users who talked about artificial intelligence (AI) during data analysis. Even marketing research suggests AI adoption (AIA) as the leading driver for a better customer experience. In a buyer–supplier relationship, AI adoption could help in the procurement process and track complaints raised by buyers (Allal-Chérif et al., 2021). AI could help buyers identify the opportunity to bring bottom-line impacts and improve internal functionalities, influencing the buyer experience. Therefore, we hypothesize:

*H7: Supplier’s AI adoption positively influences buyer experience with MSMEs.*

#### Management capabilities

The dynamic capability approach mainly draws on RBV and argues that enterprises must restructure their available resources to react to new surroundings, or collapse. RBV might describe resource distribution; meanwhile, the TCT (Brouthers & Nakos, 2004) perspective explains resource value, arrangement, and coordination. In this context, we first consider financial stability, which infers a state where a business can sustain economic shocks and provide its offering smoothly, even with its essential function (Lin et al., 2018). For a sustainable buyer–supplier relationship, financial stability is one of the key factors as it reduces management risks and intermediates third-party financial providers. However, maintaining better financial stability is a challenge for many MSMEs as they face financing constraints and have trouble getting optimal resources for their business functionalities, which is generally not an issue for the more prominent firm, especially during a crisis or global pandemics (Brei et al., 2020; Queiroz et al., 2020) like COVID-19. So, MSME suppliers with better financial stability may provide a better experience to their buyers to fulfill the designated time assignment. Also, financial stability helps suppliers cater to their customers better as financial problems make entrepreneurs show negative emotional expressions (Yoo et al., 2019). Therefore, we formulate the next hypothesis as follows:

*H8: Financial stability positively influences buyer experience with MSMEs.*

Further, TCT indicates two costs (Brouthers & Nakos, 2004), which are control and market transaction costs. By integrating its business activities, a firm may secure its unique know-how and reduce financial transaction costs. Moreover, it must combine consolidation with the expenses of regulating the hierarchical system while maintaining operational flexibility. Flexibility (FY) in operation refers to when suppliers can respond to buyers’ demand and fluctuation from within the supply chain (Chan et al., 2017). Sometimes, buyers increase the demand for raw materials from the supplier or, in some situations, decrease their order due to not consuming the previous primary products. Under such conditions, suppliers could build a positive relationship by actively monitoring the buyers’ demand behavior and providing FY in operation. However, FY requires proactive monitoring of market orientation, acquisition, and assimilation of customer information (Celuch & Murphy, 2010), which is more optimal for MSMEs due to their smaller size and close line of communication with their customer than the larger firms. Such imperatives can help SMEs implement strategic advantages. Further, borrowing the relation between FY and customer experience from service literature, which considers FY as one of the dimensions of experience (Medberg & Grönroos, 2020), we propose our next hypothesis as follows:

*H9: Flexibility in operation positively influences buyer experience with MSMEs.*

Process innovation (PI) infers the creative and improved way of processing business functionalities to increase production while decreasing costs (Lee & Schmidt, 2017). Research suggests that PI plays a significant role in value creation in the commoditized market, as there is less opportunity for product innovation (Krolikowski & Yuan, 2017; Wagner & Bode, 2014). So, removing the risk through PI suppliers can ease the business for MSME and SME buyers. Also, through PI, a supplier can provide product information and collect demand information from buyers in a more collaborative, responsive, and participative manner (Lee & Schmidt, 2017), which may further affect the buyer experience as they get information more easily. So, we propose the following hypothesis:

*H10: Process innovation positively influences buyer experience with MSMEs.*

In their investigation, Mukherjee (2018) found that many MSMEs have barriers with less infrastructural capability, storage issues, limited cash flow, and deficiency in planning. Under crisis conditions, when demand suddenly goes up, considering MSME buyer conditions, raw materials are required with a shorter turnaround time. In such a crisis situation, if the supplier cannot provide raw materials or plans according to the crisis, SME buyers lose business from their track. For example, a report by American Express indicated the cancellation of planned purchases due to poor service provided by suppliers (Express, 2017). Second, during the pandemic (unexpected crisis), MSME suppliers faced the dual challenge of lacking human resources and working capital. If they cannot provide products to their buyer in time, this may affect the buyer–supplier relationship. So, managing the crisis and planning could help SMEs’ suppliers prevent customer churn and promote long-term customer loyalty (Burhan et al., 2021). So, we propose our following hypothesis:

*H11: There is a positive relationship between crisis management and buyer experience with MSMEs.*

Many MSMEs deal with management corruption during their external and internal supply chain integration investigations. Many employees use unfair means and resources for their own benefit, affecting the interfirm relationship (Kanyoma et al., 2018). If buyers face any situation with such an employee, there may be a chance of having a bad deal, affecting the buyer’s awareness of the problem. So, management quality (MQ) needs to be substantial to build a fairer and quality deal with buyers. So, the following hypothesis is formulated as follows:

*H12: There is a positive relationship between management quality and buyer experience with MSMEs.*

Service assurance (SA) (Pitt et al., 1995) comprises quality control, quality assurance, and service-level management. It represents how suppliers ensure that the offering meets the desired quality level as promised during contract formation. Literature suggests that firms can generate a positive reputation and sustain themselves in a competitive market by ensuring consistent quality levels in their offerings. So, maintaining quality and promising better service to the buyer could be better strategies to provide a fruitful relationship. So, we have presented the following hypothesis as:

*H13: Service assurance positively influences the buyer experience in MSMEs.*

Perceived fairness (PF) refers to an individual or collective expectation regarding fairness in the deal between two or more parties (Luo et al., 2018). In the buyer–supplier relationship, both parties expect fairness in the deal from each party (Jokela & Söderman, 2017). However, the whole relationship depends upon each party’s trust and commitment; doing contract formulation without fairness may challenge the building of a fruitful relationship. Especially for MSMEs, if they feel unfair means or contract breach, buyers may affect the buyer experience towards the supplier. Even in the SM dataset, we have found consistent user talk on fairness in the quality. So, we have presented our following hypothesis:

*H14: Perceived fairness is positively associated with the buyer experience in MSMEs.*

**Fig. 5:**
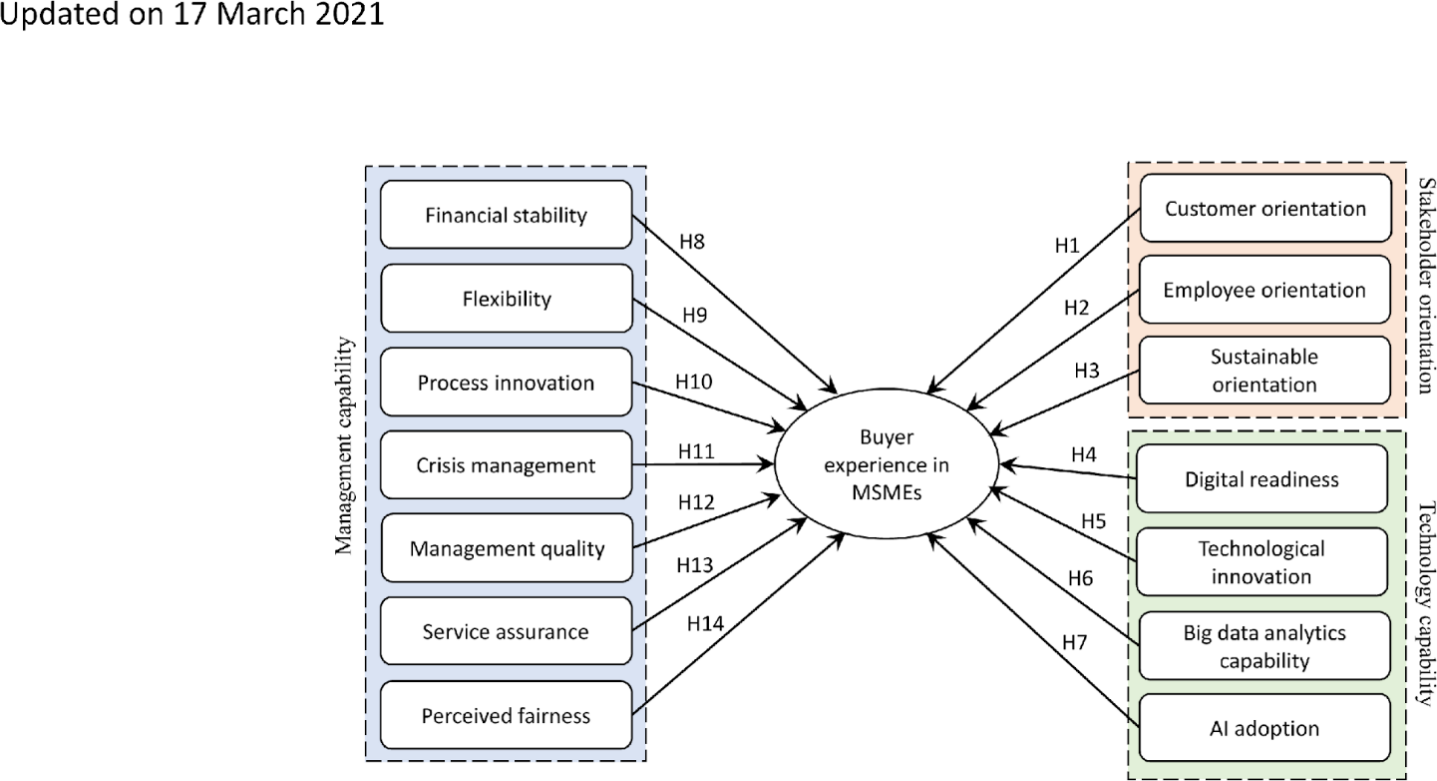
A framework to evaluate the buyer experience with MSMEs

So, the final model consisting of the factors related to buyer experience is presented in Fig.5.

### Empirical analysis

This section further verifies the relationship between independent and dependent variables using regression analysis. We have defined the target variable, leading to testing the hypotheses statements through two different models. However, the hypotheses statements remain the same across both models; it becomes essential to try these on sentiments leading to forming a polarized opinion (positive, negative, or neutral) or subjectivity scores leading to developing an emotion towards the buyer experience. We capture the contextual buyer experience through these two dependent variables portrayed by each user while writing the tweet. This contextual experience is represented by the respective models’scores (sentiment and emotion) representing experiential or satisfaction scores. Using these models establishes the drivers’ empirical relationship (hypotheses statements) with the buyer experience in each other’s presence. We conclude our analysis with one multivariate model and validate each construct’s significance in the buyer experience. First, we have presented the model’s correlation matrix (Table1) between independent variables to establish their interconnection. Table1 shows that the relationship between independent variables varies with a minor deviation expected for the direct connection (red colored box, Table1).

**Table 1:** Correlation matrix presenting the relationship between each factors

Factor	AIA	BDA	CM	CO	DR	EO	FS	FY	MQ	PF	PI	SA	SO	TI
AIA	**1**													
BDA	-0.012	1												
CM	0.000	0.001	1											
CO	0.056	0.007	0.005	1										
DR	0.023	0.006	-0.004	0.001	1									
EO	0.024	0.004	0.006	0.011	-0.006	1								
FS	0.028	-0.003	0.015	-0.008	0.000	0.058	1							
FY	-0.009	-0.003	0.002	0.002	-0.008	0.003	-0.003	1						
MQ	-0.011	-0.006	-0.024	0.000	-0.003	-0.003	-0.015	-0.003	1					
PF	-0.001	-0.004	0.003	-0.003	0.002	-0.005	0.000	-0.007	0.000	1				
PI	0.019	-0.009	0.002	0.007	0.007	-0.005	-0.006	-0.005	-0.050	0.0018	1			
SA	0.052	0.010	-0.006	0.089	0.003	0.000	0.030	-0.004	-0.001	0.0015	-0.004	1		
SO	0.006	-0.001	0.001	0.004	-0.003	-0.004	-0.003	-0.007	-0.006	-0.0017	0.001	-0.002	1	
TI	0.011	0.002	-0.001	0.059	0.003	-0.001	-0.001	0.001	0.002	0.0061	-0.002	-0.003	-0.001	1

We perform a lasso regression analysis to verify the proposed hypothesis. The 14 hypotheses have been confirmed based on the Chi-square test and p-value. We accept the hypothesis with a p-value greater than 0.05 while rejecting others. Table2 presents regression analysis results, showing that hypothesis-sounding sustainable orientation, digital readiness, technological innovation, big data analytics, AI adoption, financial stability, process innovation, crisis management, and management quality have been accepted, as their respective p-values are greater than 0.05. The hypotheses surrounding customer orientation, employee orientation, flexibility, service assurance, and perceived fairness have been rejected because their significance level is less than 0.05. So, nine hypotheses have been accepted out of 14, while five have been rejected.

**Table 2:** Coefficient and significance of variables on buyer experience in the buyer-supplier relationship

Factors	Coefficient	Standard error	p-value	Status	Result
Constant	0.1709	0.001	0.000	Significant*	Accepted
AI adoption	-0.0437	0.004	0.000	Significant*	Accepted
Big data Analytics	0.249	0.023	0.000	Significant*	Accepted
Crisis management	-0.0553	0.01	0.000	Significant*	Accepted
Customer orientation	0.0064	0.004	0.080	Non significant	Rejected
Digital readiness	-0.2792	0.024	0.000	Significant*	Accepted
Employee orientation	-0.0005	0.018	0.976	Non significant	Rejected
Financial stability	-0.0367	0.006	0.000	Significant*	Accepted
Flexibility	-0.077	0.062	0.217	Non significant	Rejected
Management quality	0.0743	0.035	0.034	Significant**	Accepted
Perceived fairness	0.0017	0.002	0.282	Non significant	Rejected
Process innovation	-6.7119	1.347	0.000	Significant*	Accepted
Service assurance	0.0125	0.011	0.232	Non significant	Rejected
Sustainable orientation	0.0303	0.019	0.013	Significant**	Accepted
Technological innovation	-0.0808	0.033	0.015	Significant**	Accepted

Significance level: * 99%, ** 95%

**Fig. 6:**
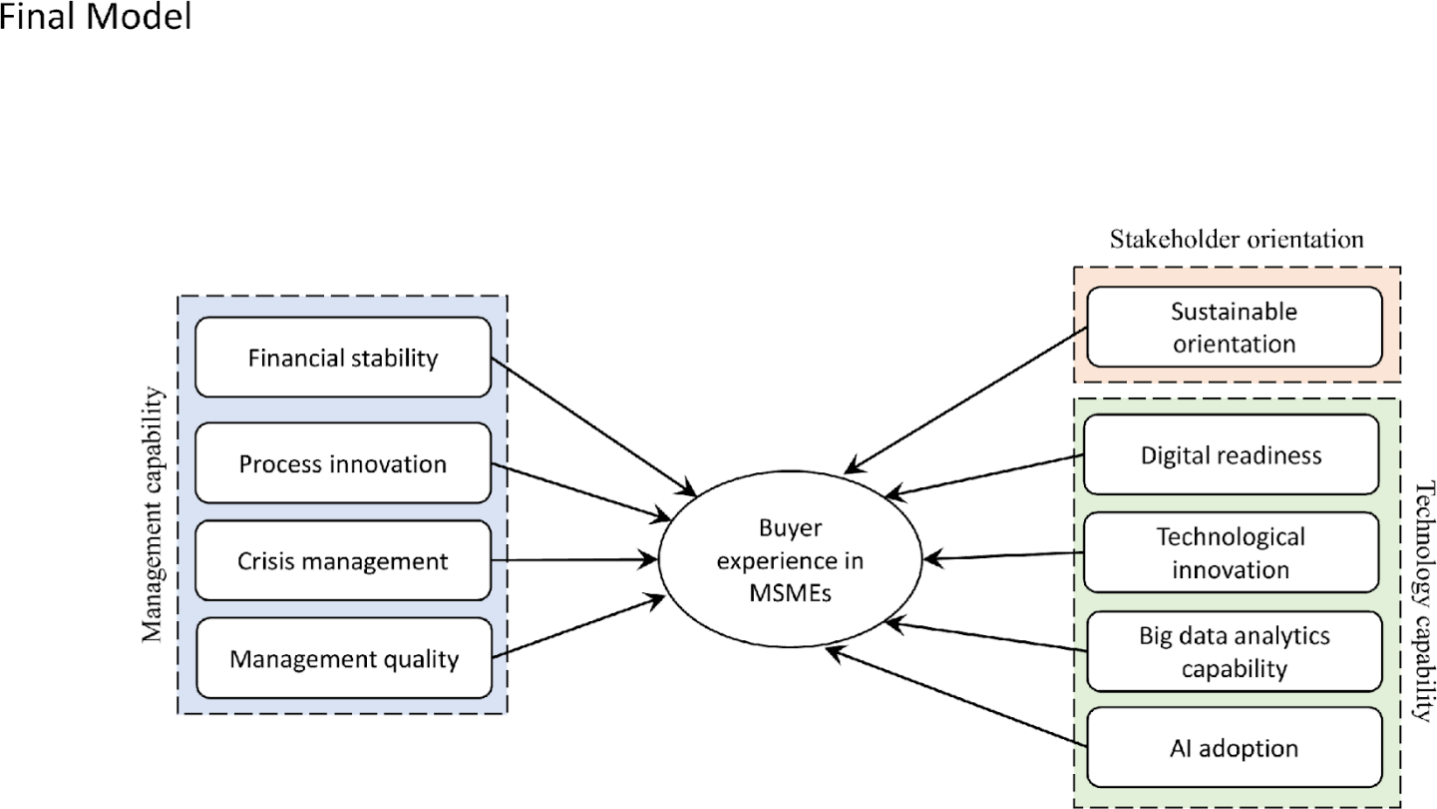
Validated buyer experience evaluation model for MSME sectors

The results support crisis management, customer orientation, digital expertise, flexibility, financial stability, innovation, perceived fairness, regulatory norms, information sharing, and sustainability orientation, while rejecting the dependency of buyer experience in MSMEs on employee orientation, management quality, process innovation, and service assurance. The final model for evaluating buyer experience after removing the rejected factors is presented in Fig.6.

## Discussion

This study aimed to extract the antecedents of buyer experience in the buyer–supplier relationship in MSMEs. We used LDA-based topic modeling on the Twitter data of 47,135 users to extract the topics, followed by applied strategies suggested by Liu et al., (2021) to map topics with the antecedents of buyer experience extracted from literature. After topic modeling, we adopted regression analysis to empirically examine the effect of these antecedents on the buyer experience. We contributed towards building a fruitful and sustainable buyer–supplier relationship in MSMEs by providing the crucial precursors of buyer experience. We have found 14 antecedents that could influence the buyer experience following our analysis. Some factors are even implied for measuring the buyer experience when considering the more prominent firms; few factors such as financial stability, flexibility, crisis management, management quality, and perceived fairness are more related to the MSME context. Out of the 14 factors, 10 show an impact relationship with buyer experience.

Moving towards this study’s second aim, to investigate the impact of extracted antecedent on buyer experience under stakeholders’ sustainable orientation (i.e., ‘climate risk’, ‘environment’, and ‘eco-friendly’) shows positive influence on buyer experience in MSMEs, which is in line with Ramirez’s (2013) analysis of customers’ views on the sustainably oriented supplier, and the perseverance of stability and a mutually beneficial relationship. Second, following literature, many MSMEs are adopting technology-associated solutions due to global crises such as the COVID-19 pandemic and worldwide lockdowns (Ali et al., 2021; Chowdhury et al., 2021). So, users on SM are talking more about different information technology (IT) solutions such as cloud-based infrastructures and video conferencing, which also impacts the buyer–supplier relationship (Azyabi, 2021). The current study on this literature expands the influence of MSMEs suppliers’ technological capability on the buyer experience. For example, results support the positive influence of AI adoption (i.e., ‘chatbot’, ‘machine learning’ ‘automatic’, ‘forecasting’) and big data analytics capabilities (i.e., ‘analytics’, ‘data science’, ‘information’, ‘assessability’) on buyer experience, indicating the reduced impact of disruption and supply chain agility (Chowdhury et al., 2021; Kushwaha et al., 2021a, b).

Studies from service literature argue that service providers’ customer orientation strategy could influence customers’ perceived performance (La et al., 2009). Moderating the customer orientation relationship with product performance followed by borrowing the concept of perceived performance from marketing literature and applying it to customers, a relationship between customer orientation and buyer experience could be established. This was reflected in Hypothesis H1, where the supplier’s focus on customer orientation could positively influence the buyer experience, but the result does not support this relationship.

Nevertheless, the relationship between employees’ orientation (i.e., ‘employee-motivation’ ‘recognition’, ‘customer-friendly’) and buyer experience is not supported (Hypothesis H2). This could be because the nature of the B2B transaction is more volatile than the B2C transaction (Tiwary et al., 2021), and the employee’s orientation concept is drawn from the internal organizational structure, which may not have a significant impact on the buyer experience. The positive relationship between crisis management (i.e., ‘lockdown’, ‘covid’, ‘pandemic’, ‘crisis-management’) and buyer experience (Hypothesis H11) was supported in terms of service context. This is not surprising as most MSMEs are not opposed to crises and pandemics. So, in a situation like the COVID-19 pandemic, if the supplier can provide their support and manage the product demand of buyers efficiently (Kurschus et al., 2017) by forecasting the order, this could build a loyal relationship with buyers.

Further, the positive influence of perceived fairness (i.e., ‘tracking’, ‘lead generation’, ‘share’) on buyer experience (Hypothesis H14) was not supported. Meanwhile, the influence of management quality on buyer experience (Hypothesis H12) was accepted. While supporting Hypothesis H8, the positive impact of the financial stability (i.e., ‘working funds’, ‘capital risk’, ‘cash-flow’) and buyer experience was also accepted. For the buyers to comprehend the supplier SMEs’ challenges and for suppliers to understand their buyers, SMEs’ preferences and motivation provide further in-depth research opportunities in this area (Chowdhury et al., 2019; Malesios et al., 2020). Our research has focused on understanding SMEs’ buyer experience and antecedents that impact the buyer experience. In many cases, buyers’ knowledge is considered customer experience, but the two are different. Likewise, the buyer experience is focused on revenue-oriented objectives and cost-effective solutions. For example, resource dependence theory (Hillman et al., 2009; Prasad et al., 2018) points to buyers’ dependency on suppliers and how it could benefit small firms. Further resource dependency creates marred touchpoints for buyer experience, and on-time resource fulfillment may provide a positive experience for the suppliers. So, providing on-time resources when buyers are in crisis creates a positive synergy in the buyer–supplier relationship (Azyabi, 2021; Kurschus et al., 2017).

### Theoretical contributions

MSMEs face many challenges during their business processes, due to which their business objective is very oriented and differs from buyer to buyer. So, understating the customer (i.e., buyer) orientation and providing them with solutions could help suppliers generate a better buyer experience (Feng et al., 2021). In the buyer–supplier relationship, the buyers’ and suppliers’ roles and strategies for maintaining a fruitful relationship are well discussed in terms of operations in supply chain management literature. What is less understood is what factors influence the buyer experience, especially when dealing with MSMEs. This study’s two critical aspects signify our contribution towards methodology to access buyer experience. First, looking toward MSMEs’ limitations in their market research, we have integrated analytical techniques and statistical methods to draw an SM-based framework to extract the buyer experience. This study integrates the antecedents of buyer experience in MSMEs through the data collected from SMP using the content analysis method. Second, this study expanded the data-driven-based inductive approach in supply chain management research beyond the factor and theme extraction using content analytics (Chae, 2015) and moved towards econometric analysis on top of content analytics for empirical validation. Further, we provide theoretical implications in the literature through content analysis and practical proof of buyer experience.

This paper indicates that RBV explains MSMEs’ strategic planning. Focusing on this procedure could provide MSMEs with competitive strategies for a better buyer experience. By examining the resource process flow more exhaustively and integrating RBV with TCT and IPT, researchers can obtain powerful and rich perspectives to enhance our comprehension of buyer experience during buyer–supplier interactions. From a contextual standpoint, we contribute towards extending the buyer experience research for MSMEs. Firstly, we considered the factors associated with three themes, stakeholder capabilities, technological capabilities, and management capabilities. Secondly, considering the MSMEs’ perspectives, we extracted four elements under these themes, financial stability, crisis management, flexibility, and perceived fairness, as essential facets of buyer experience. For example, pandemics like COVID-19 change buyer preference and behavioral patterns; under such conditions, our findings support the previous study of managers’ prior experience and accumulated knowledge to help overcome the crisis and extends to a positive association between management quality and buyer experience in the buyer–supplier relationship in MSMEs. Even the words associated with management quality, namely, ‘unprecedented planning’, ‘customer-value-proposal’, and ‘lead generation’ are consistent with previous literature on supply chains and crisis-related strategies.

Thirdly, other factors such as customer orientation, employee orientation, and management quality are also inclined towards generating buyer experience. However, larger firms have more advantages over MSMEs as they have more information acquisition and absorption capabilities. The results reinforce previous studies on suppliers’ sustainable orientation, leading to customers’ attention and indicating its influence on generating a positive buyer experience. Lastly, this research also expands supplier technology capability beyond the procurement process and technological innovation in supply chain agility, especially by confirming the positive association of big data analytics capability and AI adoption with buyer experience in MSMEs. However, the influence of digital readiness is negative on buyer experience; still, it is in line with Scuotto et al.’s (2017) findings of the negative impact of increased supply chain management costs within MSMEs.

### Managerial implications

In this research, antecedents of buyer experience are extracted through SM data, and based on existing literature, the relationship between the ancestors and buyer experience is established. First, SME suppliers face more limitations than large enterprises. So, the supplier must understand the strategies to compete in a competitive market (Zimmer et al., 2016). Suppliers can provide a better experience for their buyers, which will help them gain a long-term relationship with them and support them in building a better relationship with buyers’ peer networks for future business endeavors. The supplier needs to understand buyer expectations and the buyer’s business functionalities (customer orientation). They need to monitor the situation for when there will be a sudden demand for supply. Under such conditions, managers must adequately manage the buyers’ crisis and buy from their internals. As crisis management influences buyer experience, suppliers could forecast the demand by which they can efficiently overcome the problem (Kurschus et al., 2017).

Literature suggests that buyer–supplier relationships are often stressed due to contract breaches or the self-motivation of any party (Hawkins & Muir, 2014; Morrissey & Pittaway, 2006). Considering this situation, managers must follow fairness during contract formulation and service fulfillment. Besides, suppliers should also carefully plan before implementing an innovative solution into their operations. If buyers are large enterprises, the supplier has a minor issue. Still, if buyers are MSMEs and are not well familiar with technological innovation, they will complain about the new implementations. So, before implementing any technical solution, suppliers’ managers should consider the issue from buyers’ points of view by efficiently operating within the supply chain. For a sustainable buyer–supplier relationship, managers should consider four factors for generating a better buyer experience: financial stability, flexibility in operation, sustainable orientation, and information retrieval capabilities.

### Limitations and Future Research Scope

This study has some limitations, which we present in three terms, and based on those limitations, we suggest possible future directions. Firstly, considering the contextual perspective, we only examined the buyer experience from the MSME’s supplier perspective. Research indicates that customers’ or buyers’ understanding of experience differs from the suppliers’ experience. So, researchers may formulate separate experience models from suppliers’ and buyers’ perspectives and compare them to find the difference in their beliefs and associated factors. Also, mapping buyers’ feedback and suppliers’ experience assessment could more accurately moderate the model.

Secondly, from a methodological point of view, we have only used data from the microblogging platform Twitter. We have not considered other SM platforms like Facebook or Instagram. In addition, we have used content analysis using LDA followed by a decision tree to showcase the relationship. The factors extracted from SM data are only mapped with buyer experience. Considering the case of MSMEs, we have specific data limitations, such as the nature of Twitter data in which most tweets do not contain geolocation; in this dataset, implementing country- or state-wise constraints is not optimal. So, county-wise data will further shape the buyer experience model in the buyer–supplier relationship due to country-wise or state-wise regulatory norms or cultural influence on MSMEs. We believe that considering different SMPs could enrich the data and associate analysis.

Lastly, some factors correlate directly or indirectly to extend this study’s limitations toward the research domain’s scope. For example, management quality may be interrelated with service assurance or employee orientation. In this paper, we did not consider these linkages. So, considering these linkages may change the significance level of the relationship of those factors (e.g., customer orientation, employee orientation, service assurance), which were found to be non-significant in this study. Also, the elements extracted during the process will be more oriented towards buyer experience and reduce the standards error.

## Conclusion

This study explores the antecedents of buyer experience for MSME suppliers using SMA from user-generated content. We have used topic modeling for factor extraction, and a decision tree for relationship formation, followed by 14 hypotheses, were formulated through existing literature. Our results suggest a significant positive impact of sustainable orientation on buyers’ experience. Further, suppliers must also improve their management quality and innovate the process more impudently as management quality and process innovation significantly affect buyer experience. Due to MSMEs’ limited capabilities, governments have introduced different guidelines to protect buyers’ rights in many countries, positively impacting the buyer experience. As businesses regularly update their channels and present innovative solutions to gain a sustainable advantage, innovation (technological innovation and digital readiness) significantly impacts buyer experience. However, our data do not support the effect of perceived fairness on the buyer experience. MSMEs are continually looking for sustainable operations by which they can maintain their business in a competitive market. So, fulfilling the objective or perception of a sustainable supply chain may create a better buyer experience.

## Footnotes

1 From this point onwards the abbreviation MSMEs are assigned for signifying both MSMEs and SMEs.
